# The impact of COVID-19 on consumers' eating and purchasing habits of agricultural products in China: key determinants and policy implications

**DOI:** 10.1186/s42269-021-00694-9

**Published:** 2022-01-09

**Authors:** Thomas Bilaliib Udimal, Zhiyuan Peng, Mingcan Luo, Yan Liu

**Affiliations:** School of Economics and Management, Southwest Forestry University, Kunming, China

**Keywords:** COVID-19, Eating, Purchasing, Agricultural products, Preference

## Abstract

**Background:**

The study looks at a changed in consumer’s eating and purchasing habits during COVID-19 period. There are several modes of transmission but transmission through food as being speculated is one area that has not been confirmed through research. The study, therefore, looks at how speculations about COVID-19 spreading through food has affected consumers' eating and purchasing habits. This study through probit model analysed how consumers' eating and purchasing habits have been influenced.

**Results:**

The result shows that age, gender and education have negatively influenced consumer’s eating and purchasing habits during the COVID-19 pandemic compared to pre-pandemic period. The preference for imported food items, preference for frozen food, been infected or knowing someone who has been infected by the virus, and been infected through agricultural source or knowing someone who has been infected by the COVID-19 through agricultural source have negatively affected consumers' eating and purchasing habits compared to pre-pandemic period. The result, however, suggests that consumers who trust in the cold-chain food systems ability to limit the spread of the COVID-19 still maintain a positive eating and purchasing habits.

**Conclusions:**

The study provides evidence on the impact of COVID-19 on consumer’s eating and purchasing habits. Therefore, there is the need to institute proper sanitary measures, especially at cold-chain food systems to help curb the spread and also boost consumers’ confidence.

## Background

COVID-19 since its emergence in Wuhan City, China has since spread globally. The pandemic has affected every facet of human activities with lose of lives, jobs and collapsed of industries. The pandemic has since changed the usual life styles due to the recommendations made by World Health Organization (WHO) as means to curb the spread of the virus (World Health Organization 2020b). Some of these recommendations include social distancing, wearing of mask among others. Individual countries have also instituted measures such as travel restrictions to help curb the spread of the virus (World Health Organization 2020b). The pandemic has affected agriculture sector in diverse ways. Zhang et al. (2020a) noted that the COVID-19 has affected agricultural productivity in Zhejiang province, China. Zhang et al. (2020b) noted that the pandemic has affected agro-food systems negatively. The study notes that close to 46 million people lost their jobs in the agro-food systems, which is estimated at 7% of its added value. The study further notes that the recovery rate in the agro-food systems is very slow compared to the other sectors of the economy due to sluggish recovery of restaurants. Galanakis (2020) noted that COVID-19 pandemic has affected food security, food safety, and food sustainability. Despite provision of guidelines by WHO to help address the challenges face by food companies and other food safety systems (World Health Organization 2020a, 2020b; Djekic et al. 2021; Nakat and Bou-Mitri, 2020). New knowledge is constantly updated at local and international levels for food sector to overcome the impact of COVID-19 on the sector.

COVID-19 initial epidemiological investigation associated it first to "wet market" in the Huanan South seafood wholesale market in Wuhan. Some seem to suggest that COVID-19 could be transmitted through food. Jalava et al. (2019) noted that outbreak of infectious diseases usual occur through person-to-person, water, food or environmental. Chen et al. (2020) noted that most of the early COVID-19 reported cases were people who live and work close to Huanan 'wet market' hence the conclusion that the mode of transmission is through animals to human. Huanan "wet market" was officially closed down in January 2020, to help deal with the spread of the COVID-19. Even though these are assumptions, which are yet to be verified, they have caused a great distraction in agricultural supply chain due the association of COVID-19 mode of transmission to agricultural sources. It is estimated that value added attributed to agricultural sector decreased by 3.2% in the first quarter of 2020, with a reduction in the livestock output by 10%, which is attributed to the combined effects of African swine fever and COVID-19 (China Statistical Yearbook 2019).

The European Food Safety Authority (EFSA) concluded that there is no evidence to associate COVID-19 transmission to food sources Iriondo-DeHond et al. (2020). This is attributed to the inability of corona virus to survive on the food products or packaging materials (Djekic et al. 2021). Though food-human transmission is still disputed among researchers, the wide spread of information about the food-human transmission has a potential of distracting agricultural supply chain.

Agricultural value chain is highly linked to different sectors of the economy and any negative or positive news in the value chain is likely to affect other sectors through consumers' behaviour.

The virus mode of transmission includes contact with infected person. However, there are reported cases of people who have been infected in cold-chain food stores and imported frozen foods in China. Even though this has not received scientific backing, especially from the Western world, the development has a tendency to impact negatively on the consumers' behaviour, especially in China. There are a number of reported cases of people working in cold-chain food systems tested positive for the virus across China. With several reported cases of people, been infected by the COVID-19 at cold-chain stores, there is the likelihood that the development would affect consumers eating and purchasing habits. This study, therefore, seeks to look at how this development has impacted on the consumers' eating and purchasing habits.

Several factors influence the eating and purchasing habits of consumers, which include the product related factors, consumers related factors and claim related factors. The product related factors look at the production method and health image of the product (Lähteenmäki, 2013). The consumer related factors include demographic factors of the consumers, which vary sharply across regions and countries (Özen et al. 2014; Saba et al. 2010). The consumer related factors also vary across gender (Ares and Gámbaro, 2007; Bimbo et al. 2017; Nocella and Kennedy, 2012; Wills et al. 2009). Consumers demographic factors such as trust and socio-cultural factors have influence on purchasing habits (Annunziata et al. 2016; Ding et al. 2015; Hailu et al. 2009; Svederberg and Wendin 2011). Trust in the food supply system is likely to promote consumers eating and purchasing behaviour (Strijbos et al. 2016). Ding et al. Ding et al. (2015) revealed that trust in the food supply system is the main determinant of consumers' preference. Consumers’ trust in the food industry would increase their patronage of food offered (Siegrist et al. 2008). Low patronage of a particular food industry is a demonstration of lack of trust (Dolgopolova et al. 2015).

On the claim related factors, consumers consider their health when making purchase, especially consumables. Consumers consider the health risk associated with the purchase and consumption of a particular product and respond appropriately when there a positive or negative elements associated with the consumption of a particular product (Tan et al. 2016; Verbeke et al. 2009). Having a product linked to the spread of a particular disease would impact negatively on the eating and purchasing behaviour of consumers.

Most studies on COVID-19 have been limited to the mode of transmissions without looking at how various modes of transmissions have impacted on consumer’s eating and purchasing behaviour.

With reported cases of COVID-19 at some cold-chain stores across the country, there is the need to empirically look at how the development has influenced the eating and purchasing behaviour of consumers.

Also, with the claim that agricultural products from some countries testing positive for COVID-19, there is the need to examine how COVID-19 transmission “related factors” have influenced consumer’s eating and purchasing behaviour.

The seeks to examine the change in consumer’s eating and purchasing habits during COVID-19 period.

Based on this proposition, the study seeks to test the following hypotheses:

- H_1_: consumers who depend highly on import oriented frozen foods have developed negative attitude in their eating and purchasing habits;
- H_2_: countries of origin of imported frozen foods have influence on consumers purchasing behaviour;
- H_3_: consumers who depend on frozen foods have developed negative attitude in eating and purchasing;
- H_4_: consumers who have high preference for locally produced foods have not changed their eating and purchasing habits;
- H_5_: consumer’s trust in the cold-chain storage systems has positive effect on eating and purchasing behaviour;
- H_6_: individuals who have been infected or know someone who has been infected by the virus have developed negative purchasing attitude;
- H_7_: individuals who have been infected or know someone who has been infected by the virus through agricultural products have developed negative purchasing attitude;
- H_8_: COVID-19 has limited individuals visit to cold-chain food stores.

## Methods

### Data collection

To help achieve the study’s purpose, face-to-face questionnaires were administered at selected shops where they either sell local products or imported food products or both. This was after the School's Ethical Committee approved the questionnaire. The questionnaire was designed in English and translated into Chinese for easy administering (thus forward translation). The questionnaire administering took place between September and November 2020. The interviews were conducted within the vicinity of selected shops. They interviewed people who were either going into the shops to make purchase or exiting after making purchase. Four hundred and sixty consumers were interviewed. There was backward translation of the questionnaires after the data gathering process was completed.

Table 1 provides a list of variables considered in the study and their mode of measurement.

**Table 1:** Variables description

Age	Number of years
Gender	1 male, 0 otherwise
Education	Number of years schooling
Preference for imported products	1 if preference for imported frozen foods changed because of COVID-19, 0 otherwise
Concerned about the countries of origin	1 if concerned about the origin of imported frozen foods because of COVID-19, 0 otherwise
Has your preference for frozen food changed since the emergence of COVID-19?	1 if you still like buying frozen foods during COVID-19, 0 otherwise
Preference for local products	1 if preference for local frozen foods changed because of COVID-19, 0 otherwise
Has you or someone you know been infected by the virus?	1 if you or someone you know has been infected by COVID-19, 0 otherwise
Has your eating behaviour changed because of COVID-19?	1 if you now prefer to cook due COVID-19, 0 otherwise
Have you stopped visiting to cold-chain stores?	1 if you continue to visit cold-chain food stores despite COVID-19, 0 otherwise
Has COVID-19 changed your eating and purchasing habits?	1 if you are now careful about where and type of food products you purchase because of COVID-19, 0 otherwise
Has you or someone you know been infected through agricultural products?	1 if you or you know someone was infected by COVID-19 through food products, 0 otherwise
Do you trust the cold-chain food storage system?	1 if you trust the cold-chain storage system ability to prevent the spread of COVID-19, 0 otherwise

### Empirical model specification

The probit model, a binary classification model in which the conditional probability of one of the two possible realizations of the output variable is equal to a linear combination of the inputs, transformed by the cumulative distribution function of the standard normal distribution is adopted for the study.

With the probit model, it is assumed that a sample of data $\left({y}_{i},{x}_{i}\right),$ for $i=1,\dots ,N,$ is observed, where:

${y}_{i}$ is an output variable that can take only two values, either 1 or 0 (it is a Bernoulli random variable);

${x}_{i}$ is a $1\times K$ vector of inputs.

The conditional probability that the output ${y}_{i}$ is equal to 1, given the inputs ${x}_{i}$, is assumed to be $P\left({y}_{i=}1|{x}_{i}\right)=F({x}_{i}\beta )$

where $F(t)$ is the cumulative distribution function of the standard normal distribution and $\beta$ is a $K \times 1$ vector of coefficients.

Moreover, if ${y}_{i}$ is not equal to 1, then it is equal to 0 (no other values are possible), and the probabilities of the two values have to sum up to 1, so that

$$P({y}_{i}=0|{x}_{i}=1-P\left({y}_{i}=1|{x}_{i}\right)=1-F({x}_{i}\beta )$$

The probit model can be written as a latent variable model defined as:

1 $$ z_{i} = x_{i} \beta + \varepsilon_{i} $$

where $\varepsilon_{i}$ is a random error term having a standard normal distribution. The output $y_{i}$ is linked to the latent variable by the following relationship:

2 $$ y_{i} = \left\{ {\begin{array}{*{20}c} 1 & {{\text{if}}\,\,z_{i} \ge 0} \\ 0 & { {\text{if}}\,\, z_{i} < 0} \\ \end{array} } \right. $$

we have that

$$ \begin{aligned} & p\left( {y_{i} = 1{|}x_{i} } \right) = P(z_{i} \ge 0|x_{i} ) \\ & = P(x_{i} \beta + \varepsilon_{i} \ge 0|x_{i} \\ & = P(\varepsilon_{i} \ge - x_{i} \beta |x_{i} \\ & = P(\varepsilon_{i} \le x_{i} \beta |x_{i} \,\,\,\left( {\text{by the symmentry of the normal distribution}} \right) \\ & = F\left( {x_{i} \beta } \right) \\ \end{aligned} $$

so that the latent variable model specified by (1) and (2) assigned to the inputs the same conditional distributions assigned by the probit model.

## Result

Table 2 shows the result on the descriptive statistics. On the preference towards imported frozen foods, 70.9% of the participants indicated their preference has not changed. However, 29.1% of the research participants indicated that they have developed negative attitude towards imported frozen foods due to COVID-19. On the countries of origin, 68.7% indicated they are much concerned about the countries of origin when buying imported frozen foods due to the reported cases of COVID-19 in the imported frozen foods, 31.3%, however, indicated they are not concerned about the countries of origin when making purchase. On preference for frozen food products, 85.9% revealed that their preference for frozen foods has not changed since emergence of COVID-19, 14.1%, however, revealed a changed in preference towards frozen foods due to the reported cases of COVID-19 at cold-chain food stores. About 64.6% of the participants indicated that their preference for locally produced foods has increased due to COVID-19. About 35.4%, however, reported no changed in their preference for locally produced foods. On the trust on cold-chain food systems ability to prevent the spread of COVID-19, 40% indicated their trust in the system, 60% however, indicated lack of trust in cold-chain food system to prevent the spread of COVID-19. On infection through cold-chain food system, about 25.4% revealed that they or individuals they know were infected through cold-chain food system, 74.6% however, revealed otherwise. On change in individual’s eating and purchasing behaviour, 65.2% revealed that the outbreak of the COVID-19 pandemic has changed their eating and purchasing behaviour as they are now careful about where and what they eat and make purchase, 34.8%, however, indicated that their eating and purchasing habits have not changed since the outbreak of the COVID-19 pandemic. Concerning visits to cold-chain food stores, 68.5% indicated they have stopped visiting cold-chain food stores due to the COVID-19 outbreak, 37.5%, however, indicated that they have not stopped visiting cold-chain food stores since the outbreak of the COVID-19.

**Table 2:** Descriptive statistics

Variable	Yes	Otherwise	Total
Preference for imported products	70.9	29.1	100
Concerned about the countries of origin	68.7	31.3	100
Do you still buy frozen products?	85.9	14.1	100
Preference for local products	64.6	35.4	100
Has you or someone you know been infected by the virus?	61.7	38.3	100
Has your eating behaviour changed because of COVID-19?	62.2	37.8	100
Have you stopped going to cold-chain food stores?	68.5	31.5	100
Has COVID-19 changed your eating and purchasing behaviour habits?	65.2	34.8	100
Have you or someone you know been infected through agricultural products?	25.4	74.6	100
Do you trust the cold-chain food system?	40.7	59.3	100

*Source*: Authors’ own calculation

Table 3 presents the result on Probit regression model. The likelihood ratio chi-square of 40.55 (df = 12) with a p-value of 0.0000 tells us that our model as a whole fit significantly than an empty model (i.e. a model with no predictors).

**Table 3:** Probit regression results

Behaviour	Coef	SE	*Z*
Age	− 0.012**	0.006	− 2.11
Gender	− 0.261**	0.128	− 2.04
Education	− 0.109***	0.015	− 7.13
Imported	− 0.246***	0.103	− 3.38
Origin	0.034	0.137	0.25
Frozen	− 0.215**	0.104	− 2.07
Local	0.107	0.132	0.81
Infected	− 0.258***	0.034	− 7.56
Eating	− 0.059	0.132	− 0.44
Visit	0.337**	0.133	2.54
Agric-source	− 0.346***	0.086	− 4.01
Trust	0.507***	0.131	3.86
Constants	1.352***	0.499	2.71
Number of observations: 458			
LR chi2(12): 40.36			
Prob > chi2: 0.0001			
Pseudo R2:0.0682			
Log Likelihood: − 274.54009			

*Source*: Authors’ own calculation

Table 4 presents the result on the marginal effects. The marginal effect gives a predictive power of about 66%.

**Table 4:** Maginal effects result

Variable	d*y*/d*x*	SE	*Z*
Age	− 0.006**	0.002	− 2.11
Gender	− 0.095**	0.046	− 2.06
Education	− 0.003***	0.001	− 3.47
Imported	− 0.088***	0.029	− 3.05
Origin	0.012	0.050	0.25
Frozen	− 0.087**	0.043	− 2.04
Local	0.039	0.049	0.81
Infected	− 0.084***	0.017	− 5.01
Eating	− 0.021	0.048	− 0.45
Visit	0.126**	0.050	2.51
Agric-source	− 0.009***	0.002	− 4.50
Trust	0.179***	0.045	4.03

*Source*: Authors’ own calculation

## Discussions

The result shows that age has a statistically significant effect on individuals' eating and purchasing bebaviour during COVID-19 period compared to pre-pandemic period. Individuals who have advanced in age have developed a negative attitude in their eating and purchasing behaviour during COVID-19 compared to pre-pandemic period, and it is statistically significant at 5%. This finding corroborates earlier researches, which indicated that an increase in age leads to an increase in health related concerns, which in turn influences eating habits as they prioritize their health in their eating much than young people (Ares and Gámbaro 2007; Bimbo et al. 2017; Herath et al. 2008). Compared to pre-pandemic period, aged people have changed their eating habits. The result shows that increase in age, decreases ones eating and purchasing habits by 0.6% due to the COVID-19, it is statistically significant at 5%.

On the gender, the result shows that being male increases the likelihood of developing a negative behaviour in eating and purchasing as a result of COVID-19, and it is statistically significant at 5%. Thus, they begin to avoid some eateries and making of purchases at some food shops compared to pre-pandemic period. The result shows that males have had about 10% decrease in their eating and purchasing habits compared to their female counterparts. This is statistically significant at 5%.

The result further reveals that individuals who have spent many years schooling have developed negative attitude in eating and purchasing compared to their eating and purchasing attitude in the pre-pandemic period. They turn to avoid eating outside (at the restaurants) and making purchases at some food shops. This is statistically significant at 1%. This development can be attributed to their understanding of the COVID-19 modes of spread and access to information hence a negative attitude towards eating at restaurants and purchasing food items from some particular shops. This finding corroborates the research by Ozen et al. (2012), which concluded that education has an influence on eating habits. The result shows that consumers who have spent more years schooling have decreased their eating and purchasing habits by 0.3% due to COVID-19 pandemic compared to pre-pandemic period. This is statistically significant at 1%. More educated consumers are more responsive to the COVID-19 pandemic hence a decrease in their eating and purchasing habits.

The result shows that consumers who have high preference for imported food items have developed negative attitude in their eating and purchasing habits during COVID-19 period compared to the pre-pandemic period. This is statistically significant at 1%. Due to the COVID-19, consumers have started avoiding eating outside and buying foods items from some shops. The COVID-19 pandemic has negatively affected import foods-oriented consumers by changing their eating and purchasing behaviour. This can be attributed to the reported cases of COVID-19 in imported agricultural products, especially in cold-chain food shops. This supports H_1_ of the study. The result shows that consumers who have high preference for imported food items have had a decrease in their eating and purchasing habits by 9% due to COVID-19 pandemic. This is statistically significant at 1%. This development can be attributed to the number of COVID-19 infection cases reported at some cold-chain food stores, which mainly handles imported foods.

The result further shows that individuals who still patronize frozen foods have developed a negative attitude in eating and purchasing of food products due COVID-19. Such individuals are now more concern about what and where they eat and make less purchase now compared to pre-pandemic period. This is statistically significant at 5%. This supports H_3_ of the study. The results reveal that consumers, who indicated they still patronized frozen food items despite COVID-19, have experienced about 9% decrease in their eating and purchasing habits. This is statistically significant at 1%. This is an indication that though they still patronize frozen food items but with great carefulness hence, a reduction in eating and purchasing habits.

The result reveals that people who have been infected or know some individuals who have been infected by the COVID-19 have developed a negative attitude towards eating and purchasing food items from some places compared to the pre-pandemic period. This is statistically significant at 1%. Personal experience or knowing someone who has been infected by the COVID-19 makes individuals aware of the effect of the virus hence their negative attitude towards eating and purchasing food from some places. This supports H_6_ of the study. The consumers who have reported that either them or someone they know is/was infected by the COVID-19 experienced a decline in their eating and purchasing habits by 8%. This is statistically different from zero at (p < 0.0000). Been infected or knowing someone who is infected by the COVID-19 has caused a behavioural change in such individuals hence a reduction in their eating and purchasing habits. This finding corroborates the studies by Chrysochou and Grunert (2014) and Hooker and Teratanavat (2008), which indicated that health concerns caused a change in eating habits.

Concerning individuals' visit to the cold-chain food stores, the result shows that individuals who continue to visit cold-chain food stores despite COVID-19 have a positive attitude towards eating and purchasing of food items. This is statistically significant at 1%. It is possible such individuals are among a group of people who do not believe in the spread of the COVID-19 through cold-chain food stores. The result further suggests that individuals who still visit cold-chain food stores during COVID-19 period have a positive attitude in their eating and purchasing habits with an influence of about 13%. This is statistically different from zero. This implies that such individuals are less concern or do not believe in the assertion that the COVID-19 spreads through food items, especially cold-chain food item, hence positive eating and purchasing habits.

On the individuals who were infected or know individual(s) who were infected through agricultural source, the result shows that such individuals have developed negative attitude in their eating and purchasing of food items compared to pre-pandemic period. This is statistically significant at 1%. The result supports the study by (Bitzios et al. 2011; Tan et al. 2016), which found that claim related factors have a significance influence on consumers. Positive and negative related claims influence consumers eating and purchasing habits positively and negatively, respectively. This supports H_7_ of the study. The result shows that individuals who were infected or know someone who is/was infected by the COVID-19 through contact with agricultural products have had their eating and purchasing habits affected negatively by 0.09%. This is statistically different from zero at (p < 0.0000).

For trust, the result shows that individuals who have trust in the cold-chain food system to limit the spread of the COVID-19 still maintain a positive eating and purchasing attitude. This is statistically significant at 1%. This finding corroborates the study by Hooker and Teratanavat (2008) and Klopčič et al. (2020), which indicated that trust is key in consumers eating habits. This supports H_5_ of the study. Trust in cold-chain food systems to limit the spread of the virus increases consumers’ eating and purchasing habits by 18%, and it is statistically different from zero at 5%.

The findings corroborates earlier assertion that COVID-19 has distracted agricultural supply chain system due to the change in consumers' behaviour attributable to the association of COVID-19 with food-human transmission even though still unconfirmed (da Silva Júnior et al. 2019; Duda-Chodak et al. 2020).

## Conclusion

The study looks at changed in consumers’ eating and purchasing habits of agricultural products during the COIVD-19 pandemic period. To achieve the objective, 460 consumers were interviewed. The probit model was adopted for the analysis as the dependent variable was measured in the binary form.

Even though there is no credible and direct evidence to suggest that COVID-19 is food borne but it has distracted the food industry to a large extend.

The demographic characteristics such as age, gender and education were found to have negatively influenced consumers eating and purchasing habits during the COVID-19 pandemic compared to pre-pandemic period. Imported food consumers and frozen foods consumer were also found to have had a change in their eating and purchasing habits, as they were now careful as to where they eat and make purchases. Consumers who have been infected or know someone who has been infected by the COVID-19 have had a change in eating and purchasing habits with regards to where and what they eat and purchase. Individuals who were infected through contact with food (agriculture source) or know someone who contracted the COVID-19 through contact with food have since changed their eating and purchasing habits.

However, the result shows that consumers who have trust in the cold-chain food systems to limit the spread of COVID-19 still maintain a positive eating and purchasing attitudes. Consumers who continue to visit cold-chain food stores despites some reported cases of COVID-19 at cold-chain food systems have not had a change in their eating and purchasing habits.

From these findings, it can be concluded that the COVID-19 has negatively affected the eating and purchasing habits of consumers, the development which has the potential of affecting food industry negatively.

Although, corona virus transmission through food-human still remains speculative but based on consumers' behaviour from the current study and other modes of transmission, we, therefore propose the following for policy consideration to help deal with the current pandemic and futures ones and their effect of agricultural supply chain:

- The system to promote and enforce personal hygiene measures through the provision of refresher courses training on food hygiene principles should be encouraged at all levels of agricultural supply chain. Training on food packaging and preservation would help reduce the level of virus contamination and reduce the spread.
- For short-term and long-term measures, the use of Personal protective equipment (PPE), such as masks and gloves, should be mandatory in agricultural food supply chain.
- All segments of agricultural supply chain should strictly adhere to physical distancing and stringent hygiene and sanitation measures.
- Hand hygiene and strict adherence to the respiratory etiquette and environmental cleaning and disinfection should be encouraged in food industry.
- Hand washing facilities with sanitizers should be installed at food procession and marketing centres for patrons to wash their hands when they visit.
- In the long term, food-packaging materials that limit the spread of viral diseases should be encouraged.

## Data Availability

The datasets generated during and/or analysed during the current study are available from the corresponding author on reasonable request.

## Abbreviations

EFSA: The European Food Safety Authority
WHO: World Health Organization
